# Adhesion of Immunoglobulins to Band3 Promotes Increased Erythrocyte Sedimentation Rate in Multiple Myeloma

**DOI:** 10.1111/cpr.70149

**Published:** 2025-11-27

**Authors:** Sicheng Bian, Jiangxia Cui, Xialin Zhang, Chongzhi Bai, Yanhong Tan, Zhuanghui Hao, Xingpeng Bu, Changxin Qu, Lili Sun, Leilei Lin, Qi Wang, Zhengrui Li, Xufeng Huang, Hengrui Liu, Ruo Wang, Yinghua Li, Hongwei Wang

**Affiliations:** ^1^ Department of Medicine, The MetroHealth System Case Western Reserve University Cleveland USA; ^2^ Department of Hematology, The First Affiliated Hospital Harbin Medical University Harbin China; ^3^ Institute of Hematology The Second Clinical Medical College of Shanxi Medical University Taiyuan China; ^4^ Department of Hematology The First Hospital of Shanxi Medical University Taiyuan China; ^5^ Shanxi Academy of Advanced Research and Innovation Taiyuan China; ^6^ Central Laboratory, Shanxi Province Hospital of Traditional Chinese Medicine Taiyuan China; ^7^ Department of General Medicine, Shanxi Bethune Hospital, Tongji Shanxi Hospital Third Hospital of Shanxi Medical University Taiyuan China; ^8^ Department of Preventive Medicine The Second Hospital of Huairou Beijing China; ^9^ Department of Hematology, Affiliated Yantai Yuhuangding Hospital Qingdao University Yantai China; ^10^ Shanghai Jiaotong University School of Medicine Shanghai China; ^11^ Department of Biochemistry Cambridge University Cambridge UK; ^12^ Shengli Clinical Medical College of Fujian Medical University, Department of Breast Surgery, Fujian Provincial Hospital Fuzhou University Affiliated Provincial Hospital Fuzhou China

## Abstract

Decreased sialic acid increases the adhesion of RBC membranes to immunoglobulins leading to an increased erythrocyte sedimentation rate (ESR). The increase in reactive oxygen species (ROS) damages the sialic acid glycosyl chains on the surface of RBC membrane proteins, causing the membrane proteins to be overexposed to the plasma environment due to the loss of sialic acid coverage. Immunoglobulins in plasma adhere to RBC membrane Band3 extracellularly exposed peptides through intermolecular interactions. The reduction of sialic acid causes a weakening of the RBC membrane negative charge barrier and the adhesion of immunoglobulins further destabilises the suspension of RBCs, resulting in a rapid addition of ESR to multiple myeloma.

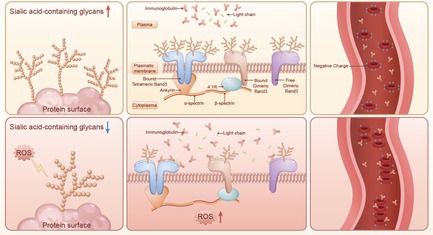

To the Editor,

Erythrocyte sedimentation rate (ESR) is a widely used metric in the clinical diagnosis of many diseases and is very sensitive to inflammation. Increased ESR has been observed in close to 85% of patients with multiple myeloma (MM) [1, 2]. The explanation for the increased ESR is often attributed to the clonal plasma cells of myeloma that secrete immunoglobulins that adhere to RBCs, but this explanation is only descriptive and speculative [3]. Although high levels of IgG may bind to RBCs through nonimmune interactions, the exact mechanism was not clear [4, 5]. The existing research is inadequate for explaining whether there are interactions between other subtypes of MM (IgA, IgD, IgM, Kappa/κ, and Lambda/λ) and RBCs, yet these subtypes continue to exhibit a high percentage of ESR acceleration.

The RBCs in the patient develop a state of peroxidative stress, which is associated with the presence of pathological hypercalcemia triggering an increase in reactive oxygen species (ROS) in MM RBCs [6]. The abnormal increase in ROS has a damaging effect on the sialic acid (*N*‐acetylneuraminic acid) glycosyl chain groups on the surface of glycoproteins [7]. Additionally, the exposure of membrane proteins due to the reduction of sialic acid may increase the interaction of plasma immunoglobulins with RBC membrane proteins, which eventually leads to changes in the ESR [3].

Therefore, elucidating the interactions between immunoglobulins and RBC membrane proteins in MM will not only be helpful in explaining the mechanism of ESR acceleration in MM, but will also promote our understanding of the pathophysiological changes that occur in ESR. It will also support further investigation of the causes of myeloid dysplasia due to MM and will provide important insights into the future development of targeted protection of normal RBCs and clearance of RBC‐bound immunoglobulins.

In a retrospective analysis of 417 cases of MM with ESR at the first visit, the incidence of increased ESR was determined to be approximately 90.89% (> 15 mm h^−1^ in male and > 20 mm h^−1^ in female) (Figure S1A). Increased ESR was observed in all types of MM, including IgG, IgA, IgD, IgM, Kappa, and Lambda. Although the prevalence of non‐secreted type MM is low, it also exhibits a high percentage of increased ESR.

The corresponding types of immunoglobulins were detected using Western blotting (WB) on the surface of peripheral blood RBCs of all types of MM involved in this study (Figure S1B). A small amount of IgG could also be detected on the surface of RBC membranes in normal controls, but none of the other types of immunoglobulins were detected in normal controls. (*P*_G_ = 0.00281, *P*_A_ = 9.25E−4, *P*_D_ = 0.00315, *P*_M_ = 5.35E−8, *P*_κ_ = 0.00138, *P*_λ_ = 0.00199) (Figure S1C) (reproduced from Zhang et al., Blood 142 (2023): Supplement 1, with permission from Elsevier. https://www.sciencedirect.com/science/article/abs/pii/S0006497123131788).

Based on the sequencing results of each co‐precipitated band, the relative expression score was calculated using GAPDH as an internal reference to screen the types of immunoglobulins adhering to the RBC membrane of each type of MM (Figure 1A,B). Co‐IP tandem protein mass spectrometry was used to analyse the RBC membrane protein molecules bound by immunoglobulins in each type of MM [8]. The results showed the presence of 16 co‐occurring protein molecules in each type of multiple myeloma samples (Figure 1C, Table 1). Eight of these protein molecules were expressed in erythrocytes and Band3 was the only RBC transmembrane protein with an extracellular peptide segment structure. The other seven protein molecules were cytoplasmic side cytoskeletal proteins of the cytoplasm/cell membrane.

**FIGURE 1:**
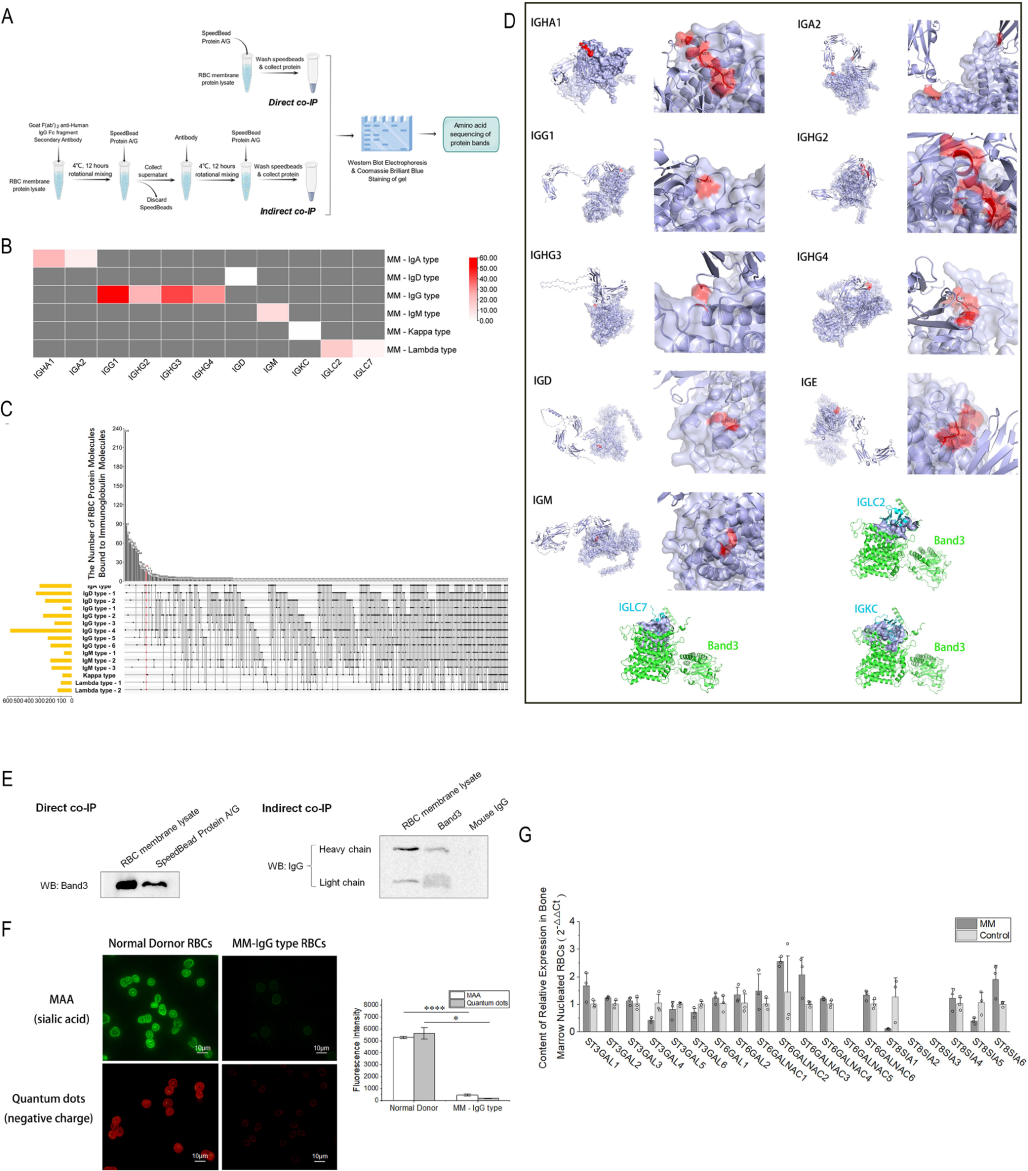
Molecular characterisation of IgG‐Band3 binding and membrane sialic acid modulation. (A) Model flow chart for direct co‐IP and indirect co‐IP related trials. (B) MM RBC membrane protein samples for detection of adherent immunoglobulin types. GAPDH was used as an internal reference to calculate the relative expression score to obtain the immunoglobulin types for various types of MM RBC adhesion. (C) MM RBC membrane protein samples for detection of immunoglobulin‐adherent RBC membrane proteins. Among the 16 RBC membrane precipitated proteins obtained, Band3 protein was the only transmembrane RBC membrane protein possessing an extra‐erythrocytic membrane peptide. (D) Various types of immunoglobulins obtained by immunoprecipitation screening were able to interact intermolecularly with band3. The intermolecular interactions were mainly intermolecular stacking interaction and hydrophobic interaction (although no IgE type multiple myeloma samples were available, we also performed molecular docking simulations with Band3). (E) Immunoprecipitation to identify the interaction between IgG and RBC membrane Band3. The presence of binding between IgG‐type MM RBC membrane Band3 and IgG was determined by Direct co‐IP and Indirect co‐IP. (F) The content of membrane sialic acid and negative charge of IgG‐type MM RBCs was measured. Compared to normal RBCs, the content of membrane sialic acid was significantly reduced in MM RBCs, and amino water‐soluble quantum dots labelled with negative cell membrane charge were also shown to be reduced in multiple myeloma RBCs. (G) The relative expression of each type of sialic acid transferase was detected in MM bone marrow nucleated RBCs. The samples were obtained using bone marrow nucleated erythrocytes by sorting, and the results showed no difference in the expression of each type of sialic acid transferase between the myeloma group and the normal control group. (Error bars represent the mean ± SEM, *p < 0.05, ****p < 0.0001. Two‐sample *t* test was used for statistical analysis).

**TABLE 1:** Screening for MM RBC membrane proteins that interact with immunoglobulins.

Uniprot	Protein	Subcellular location	Tissue specificity
P02730	Band 3 anion transport protein	Multi‐pass membrane protein	Erythrocyte
P16157	Ankyrin‐1	Cytoplasmic surface of erythrocyte membranes	Erythrocyte
P16452	Protein 4.2	Cytoplasmic surface of erythrocyte membranes	Erythrocyte
P02549	Spectrin alpha chain	Cytoplasm/cytoskeleton	Erythrocyte
P11277	Spectrin beta chain	Cytoplasm/cytoskeleton	Erythrocyte
P60709	Actin, cytoplasmic 1	Cytoplasm/cytoskeleton	Erythrocyte
P04406	Glyceraldehyde‐3‐phosphate dehydrogenase	Cytoplasm/cytoskeleton	Erythrocyte
P69905	Haemoglobin subunit alpha	Cytosol	Erythrocyte
P35908	Keratin, type II cytoskeletal 2 epidermal	Cytoplasm	Epidermis
Q86YZ3	Hornerin	Cytoplasmic granule	Epidermis
P13645	Keratin, type I cytoskeletal 10	Cell surface/cytoplasm	Suprabasal cell layers/lung cell lines
P04264	Keratin, type II cytoskeletal 1	Cell membrane/cytoplasm	Neonatal foreskin
P35527	Keratin, type I cytoskeletal 9	Cytosol/nucleus/extracellular space	Epidermis
P13647	Keratin, type II cytoskeletal 5	Cytoplasm	Corneal epithelium
P02768	Albumin	Secreted	Plasma
P01859	Immunoglobulin heavy constant gamma 2	Secreted/cell membrane	B lymphocytes

Analysis of the amino acids in the extracellular region of Band3 revealed the presence of binding surfaces capable of interacting with these immunoglobulins. The interactions between Band3 and immunoglobulins include intermolecular stacking interaction and hydrophobic interaction. For IgG, IgA, IgD, IgM, and IgE, the interaction interface exists in the extracellular segment of Band3, while the interaction between the light chain λ and Band3 is concentrated in the transmembrane region, and the light chain κ has both extracellular and transmembrane segments of Band3 (Figure 1D).

The adhesion relationship between RBC membrane Band3 protein and IgG in IgG− type MM was verified by immunoprecipitation and WB. The results demonstrated the existence of mutual binding between the two. Direct immunoprecipitation was used to directly pull down IgG in the lysate. Indirect immunoprecipitation was used to pull down Band3 protein (Figures 1E).

FITC**−**conjugated MAA ( *Maackia amurensis* lectin) was applied to label sialic acid on the surface of RBC membranes, and the fluorescence intensity was used to represent the amount of sialic acid in RBC membranes (Figure 1F). The results showed a significant decrease in the RBC membrane sialic acid content in patients with MM compared to healthy controls (*p* = 1.111E−6). The results also showed a significant decrease in the content of negative charges on the surface of RBC membranes in patients with MM compared to healthy controls (*p* = 0.0333) (Figure 1F).

Bone marrow fluid from MM and healthy controls was sorted by flow cytometry to obtain 10^6^ precursor nucleated RBCs. Total RNA was extracted from the nucleated RBCs of MM patients and of healthy controls, and cDNA was obtained by reverse transcription. q‐PCR was performed to detect the expression of 20 common human sialic acid transferases, and GAPDH was chosen as the internal reference. The results showed no difference in the expression of the 20 sialic acid transferases in precursor nucleated RBCs between the MM group and the healthy control group (Figure 1G, Table S1).

Compared to control untreated RBCs, RBCs incubated with either NA or T‐BHP showed a significant reduction in MAA fluorescence intensity (*P*_NA_versus_control_ = 0.0143, *P*_T‐BHP_versus_control_ = 0.00250). At the same time, the application of quantum dots to verify the negative charge content of RBC membranes similarly showed a significant reduction in erythrocytes after NA or T‐BHP treatment incubation (Figure 2A) (*P*_NA_versus_control_ = 0.00329, *P*_T‐BHP_versus_control_ = 0.00330). RBCs treated with two methods exhibited increased adherence to IgG (*P*_NA+IgG_versus_NA_ = 0.00181, *P*_T‐BHP+IgG_versus_T‐BHP_ = 0.00478). When human IgG/FITC was used instead of human IgG to incubate RBCs treated with NA or T‐BHP, the fluorescence values detected by the multifunctional microplate detector (the results were corrected for the protein content of the samples) revealed that both NA‐ and T‐BHP‐treated RBCs, accompanied by a decrease in sialic acid content were able to increase IgG adhesion to RBC membranes (Figure 2B) (*P*_NA+IgG_versus_NA_ = 3.85E−9, *P*_T‐BHP+IgG_versus_T‐BHP_ = 3.83E−4). An increase in ESR was observed after application of NA or T‐BHP treatment of RBCs to remove sialic acid. The increase in ESR was more pronounced after continued incubation with the addition of human IgG. Also, mild hemolysis could be observed in the RBCs treated with the application of NA (Figure 2C).

**FIGURE 2:**
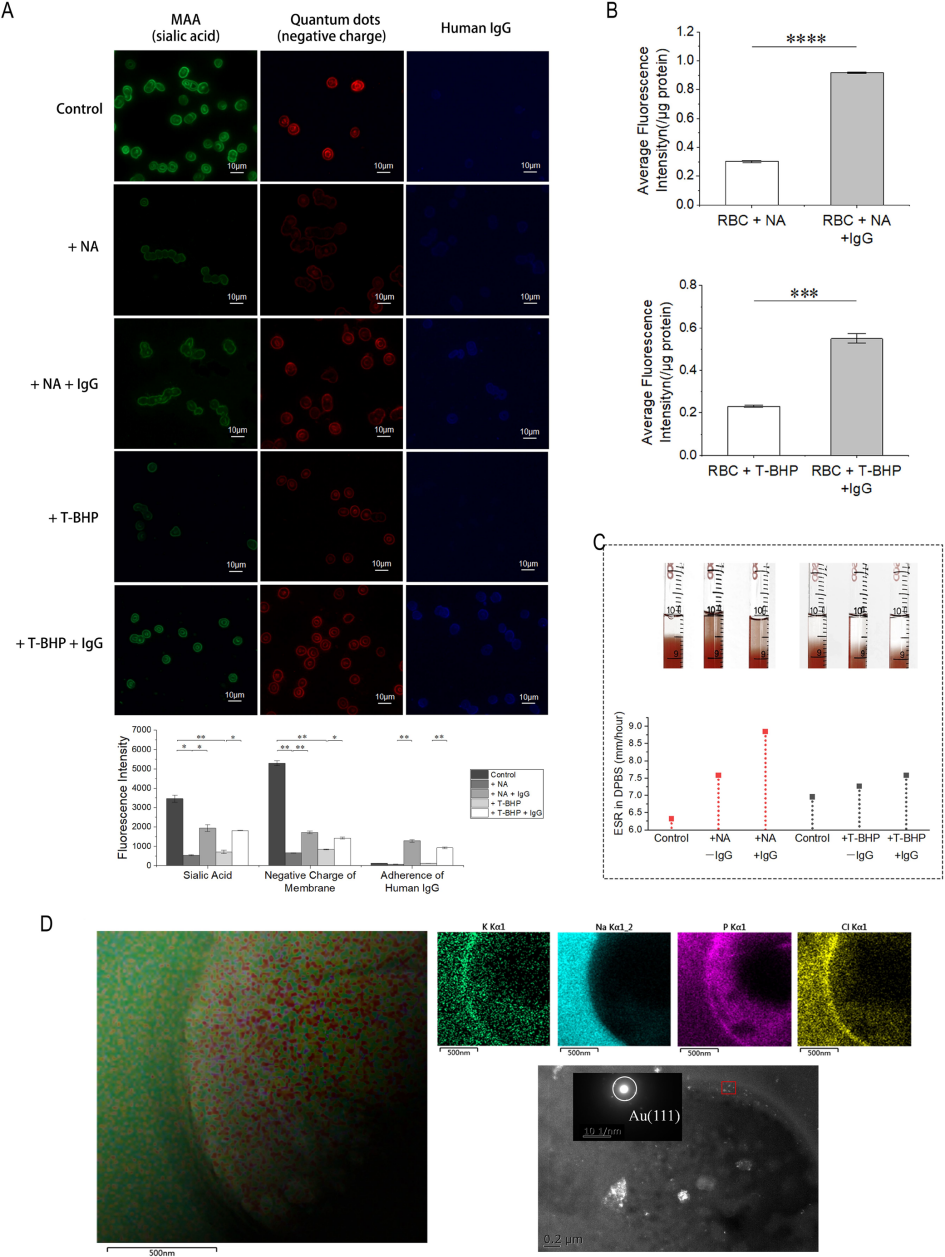
Sialic acid depletion mediates IgG adhesion potentiation and erythrocyte sedimentation acceleration. (A) The content of sialic acid affects the distribution of negative charges on the surface of RBC membranes and the adhesion of IgG. The addition of Neuraminidase (NA) to RBCs significantly reduced the amount of sialic acid in RBC membranes and increased the adhesion of human immunoglobulin IgG to RBCs from which sialic acid had been removed, a phenomenon that also occurred in T‐BHP‐treated RBCs. The destruction of sialic acid was accompanied by a reduction in the red fluorescence of quantum dot labeling on the surface of the RBC membrane showing a negative charge. When IgG adhered to the RBC membrane with sialic acid removed, there was an increase in the overall quantum dot red fluorescence of RBCs due to the inherent sialic acid on the surface of IgG. (Error bars represent the mean ± SEM, *p < 0.05, **p < 0.01, and ***p < 0.001. Two‐sample t‐test was used for statistical analysis). (B) Fluorescent enzyme assay to detect the effect of removing sialic acid from RBCs adhering to IgG. Both NA and T‐BHP were able to increase human IgG adhesion by removing RBC membrane sialic acid. (C) IgG adhesion affects the changes in ESR. The ESR of RBCs with sialic acid removed in DPBS tended to increase, and adherence to IgG could make the ESR increase further. Hemolysis could be observed in NA‐treated RBCs, indicating that the stability of RBC membranes appeared to be diminished by the disruption of sialic acid. (D) Transmission immunoelectron microscopy was performed to observe the adhesion of IgG to the membrane of RBCs with sialic acid removed. The deposition of immunogold‐labelled human IgG was able to be observed after incubation of human IgG with sialic acid‐removed RBCs. The localization of the cell membrane lipid bilayer was determined by the identification of K, Na, Cl, and P elements inside and outside the RBC membrane (error bars represent the mean ± SEM, ***p < 0.001, and ****p < 0.0001. Two‐sample *t* test was used for statistical analysis).

Normal RBCs were treated with NA and incubated with human IgG after removing sialic acid from the surface of RBC membranes. By labeling anti‐human IgG primary antibody and secondary antibody coupled with gold particles, transmission electron microscopy could observe more adhesion of gold particle–labelled human IgG adhering to the surface of RBC membranes with sialic acid removed. Further, the distribution of kalium (K), natrium (Na), chlorine (Cl) and phosphorus (P) elements inside and outside the RBC membrane was determined by elemental scanning of the observed objects, and the localization of the lipid bilayer of the cell membrane was determined (Figure 2D).

ESR is widely used in the monitoring of clinical disease as a sensitive indicator of MM disease status [4, 5]. However, the mechanism of ESR increase has not been clearly explained. Although it is widely believed that ESR acceleration in MM is by default caused by immunoglobulin adhesion to RBCs [9], this view is not supported by rigorous data and cannot explain the cause of ESR acceleration in unsecreted MM. Therefore, it would be helpful to elucidate the molecular mechanism of the interaction between immunoglobulins and erythrocyte membrane proteins to understand the pathophysiological features of MM [3].

In our study, we found that all types of immunoglobulin heavy chains are able to interact intermolecularly with the extracellular segment amino acid peptide chains of Band3 protein of RBCs by immunoprecipitation tandem protein profiling with molecular docking studies. The light chain can also interact with Band3, although it is mostly located in the transmembrane region of Band3, Due to the small molecular chain of the light chain, it has been shown that peptides of this size can penetrate biological membranes, so the light chain produced by MM can also use Band3 as a binding target protein [10].

Under normal conditions, sialic acid distributed on the plasma‐side surface of Band3 can close the amino acid sites in the extracellular region of Band3, thus preventing the approach of immunoglobulins and protecting normal RBCs from recognition and attack by autoimmunoglobulins. In contrast, due to the presence of peroxidative stress damage in RBCs of MM, this leads to degradation of RBC membrane sialic acid, so that the exposed Band3 extracellular segment amino acids become recognizable molecules for immunoglobulins and light chains, which form a prerequisite for intermolecular interactions to occur. The results of co‐IP tandem protein mass spectrometry showed that the SDS‐PAGE bands of various types of MM RBC membrane proteins were accompanied by the detection of Ankyrin and 4.1R proteins, which are immobilized proteins that bind to Band3 located on the cytoplasmic side of RBCs, further demonstrating that Band3 is a transmembrane molecule that interacts with immunoglobulins [11, 12].

The large amount of sialic acid carried on the surface of RBCs enables the presence of negative charge on the outer surface of the cell membrane and maintains the suspension stability of RBCs in plasma [13]. When sialic acid is disrupted leading to overexposure of the outer surface of the RBC membrane, it causes a decrease in the negative charge of the RBC membrane surface, while the adhesion of immunoglobulins more heavily disrupts the suspension ability of erythrocytes; these effects manifest as an increase in ESR [14]. The causes of reduced sialic acid in RBCs can be classified as decreased synthesis and increased destruction. However, the expression levels of several major sialic acid synthases in bone marrow precursor nucleated erythrocytes were not significantly reduced in MM bone marrow precursor erythrocytes. It can therefore be hypothesized that the reduced sialic acid on the membrane surface of MM RBCs is associated with increased sialic acid destruction. Eguchi H. et al. in a study on the redox reflection of salivary acid found that the reactive oxygen reagent hydrogen peroxide can damage the structure of the sugar chain that forms in salivary acid [15]. The results of previous studies have shown the presence of significantly high ROS levels in the erythrocytes of multiple myeloma [6].

This study found that immunoglobulins are mainly bound to Band3 in the RBC membrane. Band3 is an important constituent protein of the RBC membrane skeleton, and its alteration affects the rigidity and deformability of RBCs [16]. Therefore, the adhesion of immunoglobulins to Band3 may have an effect on the physical properties of RBCs and promote the sedimentation of RBCs. Band3 is an important anion transporter protein in RBCs that is involved in regulating acid–base homeostasis, ATP metabolism and O_2_/CO_2_ transport within RBCs by regulating the exchange of HCO_3_^−^ and Cl^−^ across the cell membrane [17]. When ROS levels are abnormally increased and when they are active in RBCs, they can lead to impaired anion exchange in Band3, which impairs the O_2_/CO_2_ carrying function of RBCs [18]. Therefore, in MM, abnormal binding of immunoglobulins to RBC membrane Band3 may affect the ion exchange function of the Band3 protein, leading to homeostatic imbalance in RBCs [19, 20].

In conclusion, the increase in MM ESR is associated with a decrease in RBC membrane sialic acid, which not only weakens the negative electrical repulsion between RBCs but also contributes to immunoglobulin binding due to the exposure of Band3 protein, which ultimately leads to a decrease in the suspension stability of RBCs in multiple myeloma and triggers an increase in ESR. Subsequently, we will continue to study the quantitative binding affinity of the interaction between various types of immunoglobulins and Band3 by using Surface Plasmon Resonance, and determine the spatial structure of the binding of immunoglobulins and Band3 proteins with cryo‐electron microscopy. We anticipate that the research results will elucidate the deeper mechanism by which immunoglobulins affect the increase in ESR. Simultaneously, we hope to make efforts to correct this pathological phenomenon.

## Author Contributions

Sicheng Bian, Jiangxia Cui and Xialin Zhang designed and supervised the study. Chongzhi Bai and Yanhong Tan conducted 3D structural analyses. Zhuanghui Hao and Xingpeng Bu performed bioinformatics. Changxin Qu and Lili Sun planned and conducted laboratory experiments. Leilei Lin recruited case samples. Qi Wang, Zhengrui Li, Xufeng Huang, Hengrui Liu, Ruo Wang, Yinghua Li and Hongwei Wang drafted and revised the manuscript. All authors read and approved the final manuscript.

## Funding

This work was supported by the Shanxi Provincial Natural Science Foundation of China (202203021221271 and 202203021222391).

## Ethics Statement

This study was performed in line with the principles of the Declaration of Helsinki. Approval was granted by the Ethics Committee of the Second Hospital of Shanxi Medical University (Code: 2022 YX (244), Approval Date: 10/19/2022).

## Consent

The authors have nothing to report.

## Conflicts of Interest

The authors declare no conflicts of interest.

## Supporting information


**Figure S1:** Immunoglobulin adhesion characteristics in MM erythrocytes. (A) ESR data of 417 patients with primary MM. increased ESR was seen in 90.89% of MM patients, 92.53% (223/241) in men and 88.64% (156/176) in women, and 76.92% (10/13) of non‐secretory MM also showed increased ESR. (B & C) WB and cellular immunofluorescence detection of MM RBC membrane adherent immunoglobulins (Reproduced from Zhang et al., Blood 142 (2023): Supplement 1, with permission from Elsevier. https://www.sciencedirect.com/science/article/abs/pii/S0006497123131788). The corresponding type of immunoglobulin can be detected on the RBC membrane of each type of MM. (Error bars represent the mean ± SEM, **p < 0.01, ***p < 0.001. Two‐sample t‐test was used for statistical analysis).

**Appendix S1:** Supporting Information.

## Data Availability

The data that support the findings of this study are available from the corresponding author upon reasonable request.
