# Supplementary material for: Adhesion of Immunoglobulins to Band3 Promotes Increased Erythrocyte Sedimentation Rate in Multiple Myeloma
Source: Cell Prolif. 2025 Nov 27;59(3):e70149. doi: 10.1111/cpr.70149 (PMC12961554; doi:10.1111/cpr.70149)
Supplement: Supplementary file 1 — Figure S1: Immunoglobulin adhesion characteristics in MM erythrocytes. (A) ESR data of 417 patients with primary MM. increased ESR was seen in 90.89% of MM patients, 92.53% (223/241) in men and 88.64% (156/176) in women, and 76.92% (10/13) of non‐secretory MM also showed increased ESR. (B & C) WB and cellular immunofluorescence detection of MM RBC membrane adherent immunoglobulins (Reproduced from Zhang et al., Blood 142 (2023): Supplement 1, with permission from Elsevier. https://www.sciencedirect.com/science/article/abs/pii/S0006497123131788). The corresponding type of immunoglobulin can be detected on the RBC membrane of each type of MM. (Error bars represent the mean ± SEM, **p < 0.01, ***p < 0.001. Two‐sample t‐test was used for statistical analysis). [file CPR-59-e70149-s002.docx]

**
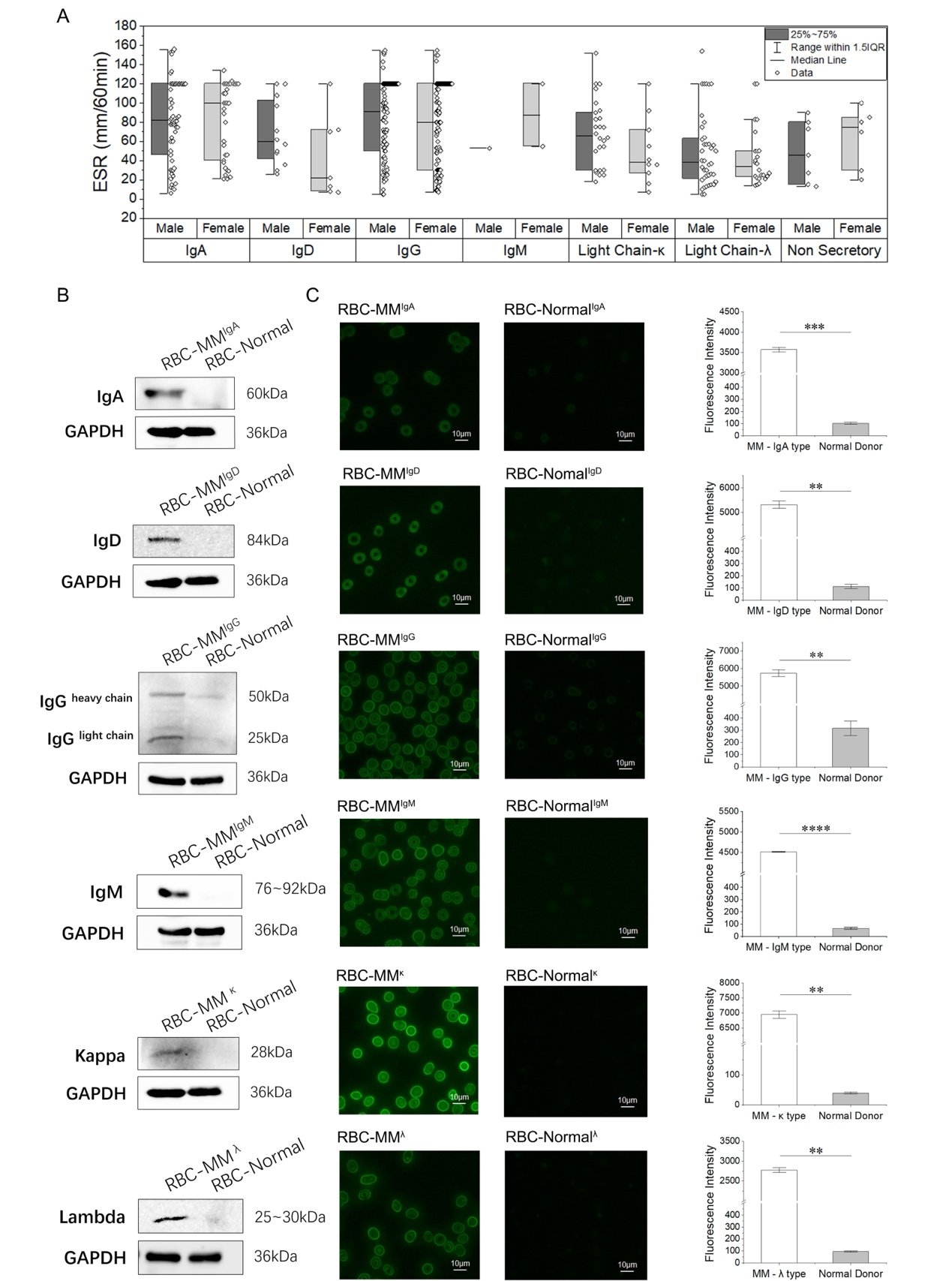
**

**Figure S1.** Immunoglobulin adhesion characteristics in MM erythrocytes.

(A) ESR data of 417 patients with primary MM. increased ESR was seen in 90.89% of MM patients, 92.53% (223/241) in men and 88.64% (156/176) in women, and 76.92% (10/13) of non secretory MM also showed increased ESR. (B & C) WB and cellular immunofluorescence detection of MM RBC membrane adherent immunoglobulins (Reproduced from Zhang et al., Blood 142 (2023): Supplement 1, with permission from Elsevier. *https://www.sciencedirect.com/science/article/abs/pii/S0006497123131788*). The corresponding type of immunoglobulin can be detected on the RBC membrane of each type of MM. (Error bars represent the mean ± SEM, ***P* < 0.01, ****P* < 0.001. Two-sample t-test was used for statistical analysis).
