# Supplementary material for: Adhesion of Immunoglobulins to Band3 Promotes Increased Erythrocyte Sedimentation Rate in Multiple Myeloma
Source: Cell Prolif. 2025 Nov 27;59(3):e70149. doi: 10.1111/cpr.70149 (PMC12961554; doi:10.1111/cpr.70149)
Supplement: Supplementary file 2 — Appendix S1: Supporting Information. [file CPR-59-e70149-s001.docx]

**Supplemental materials**

**Methods**

**Cellular immunofluorescence and chemofluorescence**

Peripheral blood RBCs were stained and then fixed with methanol for 10 min, QuickBlock™ Immunostaining Blocking Solution (P0260, Beyotime) was applied for 15 min at room temperature for nonspecific proteins, and then PBS was applied to wash the slices for 30min at room temperature. Diluted primary antibody was added dropwise and incubated at room temperature for 2 hours. PBS wash was applied for 30 min. Diluted fluorescent secondary antibody was added and incubated at room temperature for 2 hours protected from light, followed by PBS wash for 30 min. After blocking the slices, fluorescence microscopy was used for observation. *Maackia amurensis* lectin (MAA) and quantum dots were applied without dilution for chemical fluorescence labeling of cells in stock solution under incubation at room temperature and were protected from light for 2 hours. Fluorescence intensity values were analyzed using Image−Pro Plus software.

The associated antibodies and dilution ratioswere as follows: FITC Conjugated *Maackia amurensis* lectin (MAA) (F-7801-2, EY Laboratories, No dilution required), Amino Water-Soluble Quantum Dots (PEG)-625 (Q4625, WUHAN JIAYUAN, No dilution required), IgG antibody (3E8) (sc-69786, Santa Cruz, 1:400 dilution), IgG antibody (IG266) [Alexa Fluor^®^ 488] (NBP2-34648AF488, Novus, 1:400 dilution), mouse anti-Human IgA-AF488 (L35215, SAB, 1:500 dilution), mouse anti-Human IgM-AF488 (L35218, SAB, 1:500 dilution), IgD antibody (IgD26) (sc-53345, Santa Cruz, 1:400 dilution), Igλ chain antibody (N10/2) (sc-53344, Santa Cruz, 1:400 dilution), Igκ chain antibody (L1C1) (sc-59265, Santa Cruz, 1:400 dilution), anti-mouse IgG (H+L) CF™ 405S antibody produced in goat (SAB4600023, Sigma–Aldrich, 1:100 dilution), goat anti-rabbit IgG H&L/FITC antibody (bs-0295G-FITC, Bioss, 1:500 dilution), and goat anti-mouse IgG H&L/FITC antibody (bs-0296G-FITC, Bioss, 1:500 dilution).

**Extraction of RBC membrane protein**

Human peripheral blood lymphocyte isolate was applied to purify the peripheral blood RBCs, and 1 ml of pure water was added to 400 μL of RBC precipitate to lyse the RBCs. Then, the samples were centrifuged at 15,000×g for 25 min at 4 °C, the supernatant was discarded, and the lower floc layer was retained. The RBC membrane precipitate was washed repeatedly with pure water until a milky white RBC membrane precipitate was obtained. Two times the volume of protein lysate (containing phenylmethanesulfonyl fluoride at a concentration of 1 mmol‧L^−1^) was added to the RBC membrane, lysed for 1 hour at 4 °C and then centrifuged at 15,000×g for 10 min at 4 °C to obtain the RBC membrane protein supernatant.

**Western blot**

Proteins were electrophoresed using a 10% SDS-polyacrylamide gel and transferred onto 0.45 μm polyvinylidene fluoride (PVDF) Membranes (03010040001, Roche) by a wet transfer apparatus. 5% Difco™ Skim Milk (232100, Becton, Dickinson and Company) for nonspecific protein closure. FluorChem FC3 was used to capture the protein fluorescence signal after enhanced chemiluminescence (ECL) incubation.

Associated antibodies and dilution ratios were as follows: Human IgG Polyclonal antibody (10284-1-AP, Proteintech, 1:2000 dilution), IgA antibody (CY6707, Abways, 1:1000 dilution), IgM antibody (R1/69) (sc-53347, Santa Cruz, 1:400 dilution), IGHD antibody (CY8568, Abways, 1:1000 dilution), Ig κ chain antibody (L1C1) (sc-59265, Santa Cruz, 1:400 dilution), Ig λ chain antibody (N10/2) (sc-53344, Santa Cruz, 1:400 dilution), GAPDH Rabbit pAb (AC027, ABclonal, 1:5000 dilution), recombinant anti-Band 3/AE 1 antibody (ab108414, Abcam, 1:5000 dilution), mouse anti-rabbit IgG-HRP (sc-2357, Santa Cruz, 1:5000 dilution), and m-IgGκ BP-HRP (sc-516102, Santa Cruz, 1:5000 dilution).

**Coimmunoprecipitation (co-IP)**

Direct co-IP was conducted as follows: In 500 μg of erythrocyte membrane protein lysate (total volume 700 μL), 10 μL of Sera-Mag SpeedBead Protein A/G suspension (17152104011150, GE Healthcare) was added, and mixed vertically for 7 hours at 4 °C. Magnetic beads were obtained after incubation from a magnetic rack, and the protein lysate was washed 5 times. Then, 30 μL of 3×Blue Loading Buffer Pack (7722, Cell Signaling Technology) containing a concentration of 100 mmol‧L^−1^ Dithiothreitol (DTT) was added, heated at 100°C for 5 min, and the supernatant that remained on the magnetic rack for 2 min was the precipitated protein sample.

Indirect co-IP was conducted as follows: Goat F(ab')2 anti-human IgG Fc fragment secondary antibody (2.8 μL) was added to 500 μg of erythrocyte membrane protein lysate (total volume 700 μL) at a ratio of 1:250 to block the Fc segment of IgG present in the erythrocyte membrane protein and to block the the myeloma-associated IgG adhering to the membrane proteins from developing an affinity with the magnetic beads in subsequent experiments. The mixture was placed in a vertical mixer at 4 °C for 12 hours. Ten microliters of prewashed Sera-Mag SpeedBead Protein A/G mix was added and mixed vertically at 4 °C for 7 hours. The supernatant protein lysate was transferred to a new Eppendorf (EP) tube after being placed in a magnetic stand and the beads were discarded. Two micrograms antibody for immunoprecipitation or normal mouse IgG was added to the lysate and mixed vertically at 4 °C for 12 hours. Ten microliters of prewashed Sera-Mag SpeedBead Protein A/G suspension was added and mixed vertically at 4 °C for 7 hours. Magnetic beads were obtained from the magnetic rack after incubation, and the protein lysate was washed 5 times before adding 30 μL of 3× Blue Loading Buffer Pack containing a concentration of 100 mmol‧L^−1^ DTT, followed by heating at 100 °C for 5 min. This was then placed on the supernatant remaining in the magnetic rack for 2 min to serve as the precipitated protein sample.

The protein precipitation samples obtained were separated by electrophoresis by applying a 10% SDS‒PAGE assay. After electrophoresis, the gels were were stained with Coomassie brilliant blue dye (P0017F, Beyotime), and the target protein bands were excised for protein mass spectrometry. The co-IP products obtained were validated by WB. All antibodies required for WB validation were detected using rabbit anti-human primary antibody (see WB experiments section).

The relevant antibodies for immunoprecipitation applications were as follows: band 3 antibody (A-6) (sc-133190, Santa Cruz), IgG antibody (3E8) (sc-69786, Santa Cruz), IgA antibody (A-9) (sc-373823, Santa Cruz), IgD antibody (IgD26) (sc-53345, Santa Cruz), IgM antibody (R1/69) (sc-53347, Santa Cruz), Igκ chain antibody (L1C1) (sc-59265, Santa Cruz), Igλ chain antibody (N10/2) (sc-53344, Santa Cruz), normal mouse IgG (sc-2025, Santa Cruz), and goat F(ab')2 anti-human IgG Fc fragment secondary antibody (Pre-adsorbed) (NBP1-75456, Novus).

**Protein‒protein docking**

Protein 3D structure prediction was conducted based on the amino acid sequences of Band3, immunoglobulin heavy chain and light chain proteins with ZDOCK 3.0.2, a holistic search docking engine based on Fourier transform technology that uses full-space search sampling to filter the closest natural conformation from the 2000 predicted structures without any binding site information. In addition to taking full advantage of the geometric complementarity between immunoglobulins and the receptor binding domain (RBD) of band3, the docking process also accounts for the contribution of electrostatic interaction energy and conformational changes between immunoglobulins and the RBD. The model with the highest ZDOCK score was chosen for the analysis.

**Sorting of nucleated RBCs**

Ficoll isolate was used to obtain mononuclear cells from bone marrow fluid of MM patients and healthy donors. CD45**‒**PC7 (IM3548, Beckman Coulter) and CD36**‒**FITC (IM0766U, Beckman Coulter) were applied to incubate the single nucleated cells for 1hour at room temperature with protection from light. The cells were washed with Dulbecco's phosphate buffered saline solution (DPBS), and the cell number was adjusted to 10^7^~10^8^ mL^−1^. CD36^+^/CD45^−^ precursor erythrocytes were then sorted by flow cytometry, and the sorted cells were subjected to flow cytometry to identify their purity.

**RNA isolation and qRT‒PCR**

Total cellular RNA was extracted using RNAiso Plus (9109, TaKaRa). cDNA was reverse transcribed using Primer Script RT Master Mix (RR036A, TaKaRa). cDNA was detected using an ABI 7500 Real**‒**Time PCR system configured by FastStart Universal SYBR^®^ Green Master Mix (ROX) (04913914001, Roche) was used to configure the amplification reaction system. Primer synthesis was performed by Sangon Biotech (Primer sequences: Supplementary Table 1).

**Construction of RBC oxidative stress**

Tert-butyl hydroperoxide (T-BHP) (458139, Sigma**‒**Aldrich) was diluted using DPBS and configured to a working concentration of 0.10 mmol‧L^−1^. Peripheral blood erythrocytes from healthy donors were incubated for 10 min at room temperature, and then the erythrocytes were immediately washed 3 times with DPBS to remove the residual T-BHP from the reaction system, and the cell precipitate was resuspended using DPBS.

For the IgG adhesion studies, 200 μg of human IgG (bs-0297P, Bioss) were added to T-BHP-treated RBCs and washed three times with DPBS after a 2 hours water bath at 37 °C. The treated RBC smears were fixed with methanol and labeled by cellular immunofluorescence.

The applied fluorescent antibodies were as follows: IgG antibody (3E8) (sc-69786, Santa Cruz) and anti-mouse IgG (H+L) CF™ 405S antibody produced in goat (SAB4600023, Sigma**‒**Aldrich). The ESR changes in treated RBCs in DPBS were be detected.

**Erythrocyte desialylation**

Neuraminidase (NA) from *Clostridium perfringens* (*C. welchii*) (N2876, Sigma**‒**Aldrich) was diluted using DPBS to obtain a working solution at a concentration of 1 U∙mL^−1^. Normal erythrocytes were incubated at 37 °C for 1 hour, followed by 3 washes in DPBS. The cellular immunofluorescence assay for IgG adhesion and the detection of ESR in DPBS were continued later.

**Fluorescence detection of RBC‒adsorbed antibodies**

Human IgG/FITC (bsF-0297P, Bioss) was added to the T-BHP or NA-treated RBCs suspension at a dilution ratio of 1:100 and incubated at 37 °C for 2 hours. The fluorescence intensity was measured at 520 nm using a multifunctional microplate tester (H1, Bio Tek Synergy) under 488 nm excitation. A Bradford assay was applied to determine the erythrocyte protein concentration of each group of samples during the experiment, and the results were used to correct the obtained fluorescence intensity.

**Immunoelectron microscopy**

After coincubation of human IgG with sialidase-treated RBCs, IgG antibody (3E8) (sc-69786, Santa Cruz) was added to the cell suspension at a dilution of 1:50 and incubated at room temperature for 2 hours. After washing the cells 3 times with DPBS, goat anti-mouse IgG H&L/Gold antibody (diameter 35 nm, bs-0296G-Gold, Bioss) was added at a dilution of 1:100 and the cells were incubated for 2 hours at room temperature. The cells were then washed 3 times with PBS and fixed with para-formaldehyde/glytaraldehyde in PBS (4%/0.5%) (B640654, Shanghai Boer). After fixation, 20 μL of the cell suspension was added dropwise onto a 200-mesh carbon-film copper mesh, dried and placed in a high-resolution transmission electron microscope (JEM-2100F, JAPAN Electron OPTICS LABORATORY) for observation.

**The role of sialic acid in the adhesion of RBC membrane to IgG test**

Peripheral blood RBCs from healthy donors were incubated with 0.10 mmol‧L−1 of tert-butyl hydroperoxide (T-BHP) (Sigma‒Aldrich) for 10 min to obtain an RBC oxidative stress model. Sialic acid-removed RBCs were obtained by incubating normal RBCs for 1 h at 37°C using 1 U∙mL−1 of Neuraminidase (NA) from Clostridium perfringens (C. welchii) (Sigma‒Aldrich). IgG incubation was performed using human IgG (Bioss) or human IgG/FITC (Bioss) incubated with T-BHP or NA treated RBCs, respectively. Cell immunofluorescence and a multifunctional microplate tester (Bio-Tek Synergy) were used for detection. A high-resolution transmission electron microscope was also used for the observation of adsorbed human IgG.

**Supplemental materials: Table 1. Primer sequences**.

| **Gene** | **Forward Primer** | **Reverse Primer** |
| --- | --- | --- |
| GAPDH | ACAACTTTGGTATCGTGGAAGG | GCCATCACGCCACAGTTTC |
| ST6GAL1 | AACTCTCAGTTGGTTACCACAGA | GGTGCAGCTTACGATAAGTCTT |
| ST6GAL2 | AAGGGGAACGTCTCTTCCAAA | CTTGTTGGCGGTCAGGTAATC |
| ST3GAL1 | ACAGTCACGACTTTGTCCTCA | TGGTCTTGGTCCCAACATCAG |
| ST3GAL2 | CGTCTGGACCCGAGAGAAC | GCCAGGCACTATCTGGAACA |
| ST3GAL3 | GCCCACTTGCGAAAGGAGT | GCCTGCTGAATTAGCCACCAA |
| ST3GAL4 | CAGTGGCTGGCTATGAGGG | GGTCGAAGTGGGCAGATTCA |
| ST3GAL5 | AGGAATGTCGTCCCAAGTTTG | GGAGTAAGTCCACGCTATACCT |
| ST3GAL6 | ATTGCCATCACATTGGCGTTT | GCAAAGGACTCTTGAGGTCAG |
| ST6GALNAC1 | CACAGCCAAGACGCTCATTC | CCTTTCTGTCTCGTCCTTGTTG |
| ST6GALNAC2 | CCTCCAAAGTGTATCCGGTGT | AGTAGGAGACGAGGGAGTTCT |
| ST6GALNAC3 | CAACTGGACTGTGACCTTTGT | GGCCGACATCTTCTTCATAACC |
| ST6GALNAC4 | TGTGAGGAGATCGTGGTCTATG | CAAAGTAGTGGTAAGGCACTGAG |
| ST6GALNAC5 | CACTGGCTGGTTTACAATGACA | GTCCTCGCTCATGGGAGAG |
| ST6GALNAC6 | ATCCGCATGAATGATGCACC | GGGGTCCGGTTGACAAACTC |
| ST8SIA1 | CATGCGATGCAATCTCCCTC | CTGGGATTAGCTGTCACTAACTG |
| ST8SIA2 | CACAGCTTCGTCATCAGGTG | GTTCATGGTTACCAGGTCTGTC |
| ST8SIA3 | ACAGCGTTTTTACATCAAAGGC | CAGTTGTCCAATCCGAACACTAT |
| ST8SIA4 | GAAAGGCTGGCTCTTCAATCT | ACCACTGACACATCTCGTTCT |
| ST8SIA5 | ACCGGGATTTGTTGGGGAG | CATACAGGATCTGTTGCAGCA |
| ST8SIA6 | CGCCACTAACAGCACATATCTG | ATCTGAAGGTAGTCGTTCTCTGA |
